# Comparison of Changes in Sterol Content of Nuts After Roasting Using Conventional and Microwave Methods and After Storage

**DOI:** 10.3390/molecules30030606

**Published:** 2025-01-30

**Authors:** Klaudia Kulik, Bożena Waszkiewicz-Robak

**Affiliations:** 1 Department of Functional and Organic Food, Institute of Human Nutrition Sciences, Warsaw University of Life Sciences, Nowoursynowska Str.159c, 02-776 Warsaw, Poland; 2 School of Medical & Health Sciences, University of Economics and Human Sciences in Warsaw, Okopowa 59, 01-043 Warszawa, Poland; b.waszkiewicz-robak@vizja.pl

**Keywords:** walnuts, hazelnuts, peanuts, microwave roasting, conventional roasting, storage, sterols, stability

## Abstract

The aim of this study was to determine the influence of the nut roasting process (conventional and microwave methods) and long-term storage (12 months) on phytosterol content and stability. This study was conducted using hazelnuts (*Corylus avellana*), common walnuts (*Juglans regia* L.), and shelled peanuts (*Arachis hypogaea* L.). Two roasting methods were examined: conventional (temp. 170 °C, roasting time 10–20 min.) and microwave (temp. 60 °C, pressure 40 hPa, roasting time 140–180 s). In the studied nuts (raw, roasted and stored), five main types of phytosterols were identified: campesterol, stigmasterol, ß-sitosterol, delta 5-avenasterol and cycloartenol. It was shown that the microwave roasting method caused a two-fold decrease in sterol loss compared with conventional roasting. Moreover, the long-term storage of roasted walnuts using the microwave method showed double the amount of sterols preserved compared with those roasted using the conventional method. The amount of ß-sitosterol, which was the most stable during roasting, depended more on storage duration than on roasting process. The cycloartenol content in the roasted nuts did not depend on storage duration. The sterols present in nuts, raw or roasted using either method, transform more during the first 6 months of storage.

## 1. Introduction

Consumers are becoming more aware of products rich in various healthy ingredients such as plant sterols. For many years, the health-promoting role of phytosterols has been emphasized, especially in the prevention of cardiovascular diseases [1,2,3,4]. Phytosterols, both in free and esterified form, reduce the risk of atherosclerosis and limit the absorption of cholesterol from food, which, in turn, makes it harder for cholesterol to accumulate in human blood [5,6,7,8]. Moreover, phytosterols can prevent various types of cancer and inflammation [9]. They can also act as biological antioxidants [6,10,11,12].

The health-promoting effect of sterols on the human body depends on their daily intake in the diet [13,14,15,16]. Therefore, it is very important to know the actual content of phytosterols in various foods, including nuts, which are increasingly recommended for consumption, also as a source of phytosterols [17].

In 2003, the Food and Drug Administration (FDA) published the first accepted health report on walnuts. It concluded that eating about 42.5 g of walnuts per day as part of a diet low in saturated fat and cholesterol could reduce the risk of heart disease. However, it was stipulated that nuts should not contain more than 4 g of saturated fat per 50 g of the product [18]. In 2008, EFSA stated that the minimum dose of phytosterols to reduce serum cholesterol (approx. 10–15%) is 1 g per day, even though scientific studies indicate a daily intake of 1.5–3 g of sterols [19]. Unfortunately, very low intake of these ingredients is observed in the Western diet, estimated at 150–400 mg/day. The vegetarian diet (800–900 mg/day) is much richer in phytosterols, but it is still far from the recommended dose. These data justify the need to enrich the diet with foods naturally rich in phytosterols. These are mainly vegetable oils, legume seeds, cereal products and nuts [20]. Increasing scientific evidence shows that tree nuts are really valuable for their nutritional, health and sensory attributes [21].

In 2011, the European Food Safety Authority issued an opinion [22] in which it indicated that eating 30 g of walnuts per day improves blood vessel dilation, which depends on the endothelium (EDV), the interior of the blood vessel.

In 2016, the EFSA published information on dietary recommendations for the 21st century. This publication indicated that the human diet should be based on a wide range of nutrient-rich raw materials. Among these raw materials, wood nuts are indicated as products of the daily diet [23]. In 2016, nuts were listed as products recommended for daily consumption in the Healthy Nutrition and Physical Activity Pyramid developed by the Food and Nutrition Institute in Poland [24].

Numerous publications confirmed the presence of phytosterols in various types of nuts or nut oils [2,8,9,25,26,27], with their influence (including those found in nuts) on the human body and the indication of consumption conditions being well described [5,10,16,28,29,30,31,32,33,34,35,36]. However, reports suggest that the stability of phytosterols in nuts during the process of storage and/or processing is not comprehensively documented.

Available scientific studies describe the effect of various technological processes on the content of plant sterols, but they mainly concern vegetable oils. This is because they are considered the best source of sterols. In contrast, relatively little research has so far been conducted on phytosterol-rich raw materials other than oils, which are subjected to various processing methods. This creates the need for extensive research indicating the content of these valuable components in raw materials and in finished products processed in different ways.

Therefore, the aim of this study was to evaluate the effect of different methods of roasting nuts (conventional and microwave) and their storage for 3, 6 and 12 months on the content of selected phytosterols.

## 2. Results

### 2.1. Stability of Phytosterols During Storage of Raw Nuts (Unroasted)

The content of selected phytosterols contained in raw nuts stored for 3, 6 and 12 months under refrigeration (+4 °C) was determined for hazelnuts, walnuts and peanuts. In all nuts tested, the following were identified: ß-sitosterol, campesterol, stigmasterol, delta 5-avenasterol and cycloartenol (Table 1).

Walnuts were characterized by a significantly higher sterol content than other types of nuts, namely, 114.07 mg/100 g. Peanuts and walnuts contained similar total phytosterols (92.73 and 91.87 mg/100 g, respectively). In all cases, the dominant type of phytosterol was ß-sitosterol.

**Figure 1:**
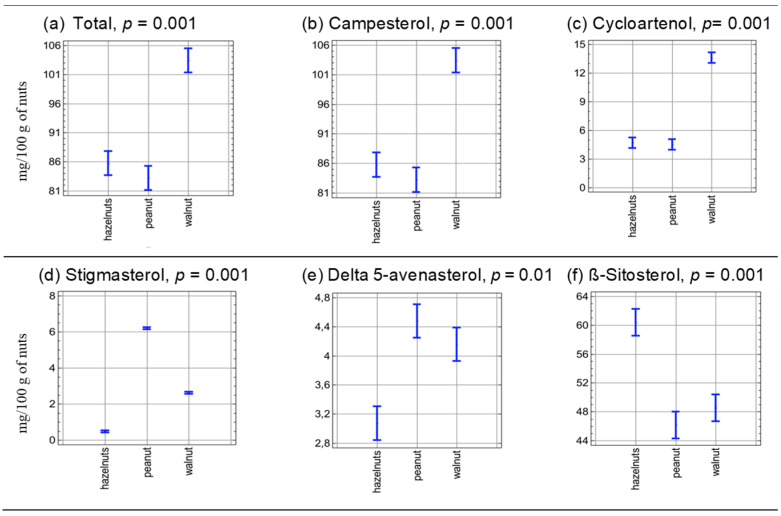
Graphical interpretation of the two-factor analysis of variance determining the effect of the type of nuts stored at different times (0 to 12 months) on the phytosterol content (MULTI-ANOVA, alpha = 0.05) (*n* = 54).

**Figure 2:**
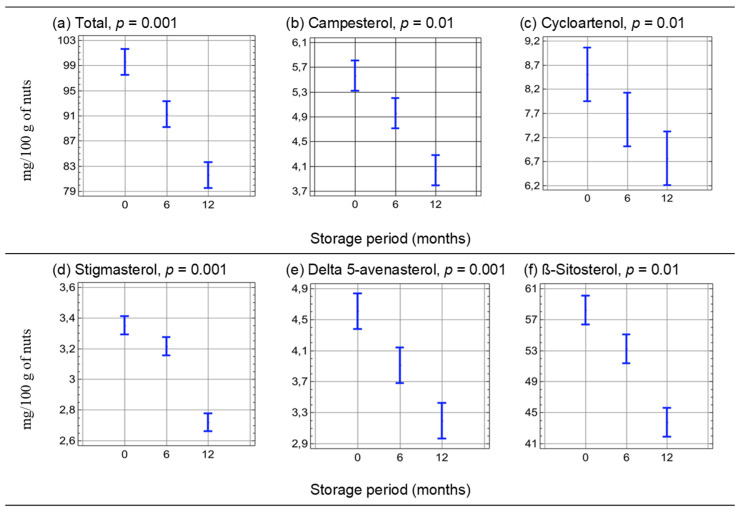
Graphical interpretation of the two-factor analysis of variance determining the effect of the storage time of the tested nuts (hazelnuts, walnuts and peanuts) on the phytosterol content(MULTI-ANOVA, alpha = 0.05) (*n* = 54).

Figure 1 and Figure 2 show the graphical interpretation of a two-factor analysis of variance indicating the influence of nut type (factor 1—Figure 1) and storage time (factor 2—Figure 2) on the content of the identified phytosterols. Peanuts (peanuts) and hazelnuts (hazelnuts) can be compared in terms of total phytosterol content, campesterol and cycloartenol. The content of other sterols differed significantly between the nut groups (*p* < 0.05).

**Figure 3:**
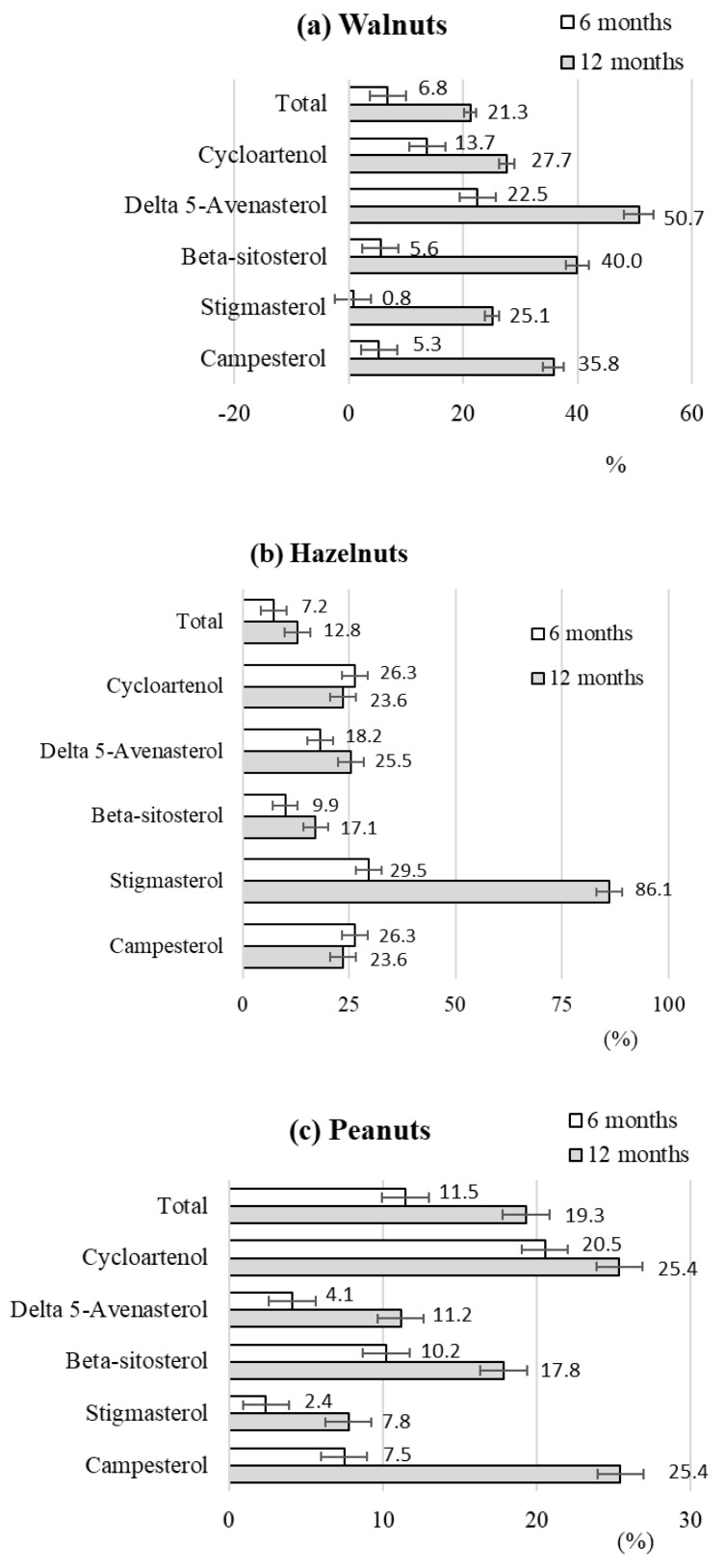
Comparison of losses of individual types of sterols during long-term storage of raw walnuts (**a**), hazelnuts (**b**) and peanuts (**c**).

During storage of the tested raw nuts, a significant decrease in the content of individual sterols was observed. Figure 3 summarizes the losses of the tested phytosterols that occurred after 6 and 12 months of storage of the tested nuts.

After 6 months of storage of the raw nuts, the total sterol loss was 6.8% for walnuts, 7.2% for hazelnuts and 11.5% for peanuts. After 12 months, these losses exceeded 21.3%, 12.8% and 19.3%, respectively. For walnuts, the highest losses were observed for delta 5-avenasterol, which was lost by 22.5% after 6 months and by 50.7% after 12 months of storage. In contrast, the lowest losses were observed for stigmasterol, the content of which remained practically unchanged during the first 6 months of storage. After 12 months of storage, 25.1% of its original content was lost (Figure 3a).

In the case of hazelnuts, the greatest losses were recorded for stigmasterol, which was lost by 29.5% after 6 months and by 86.1% after 12 months of storage. The most stable sterol in terms of content in hazelnuts was ß-sitosterol, which decreased by 9.9% after the first 6 months and by 17.1% after 12 months (Figure 3b). Peanuts had the lowest loss of stigmasterol, the original content of which decreased by 2.4% after 6 months and by 7.8% after 12 months of storage (Figure 3c).

### 2.2. The Effect of Roasting and Long-Term Storage of Nuts on the Sterol Content in Nuts

The effect of the roasting and storage process of nuts for 3 to 12 months on the sterol content was determined using walnuts collected in 2017. Two types of roasting were used in this study: conventional and microwave. Table 2 compares the total content and loss of sterols from walnuts, which were determined immediately after roasting and after different storage times. Conventional roasting reduced their total content by 13.8% compared to the original amount, while microwave roasting reduced the original amount of sterols by only 7.3%.

**Figure 4:**
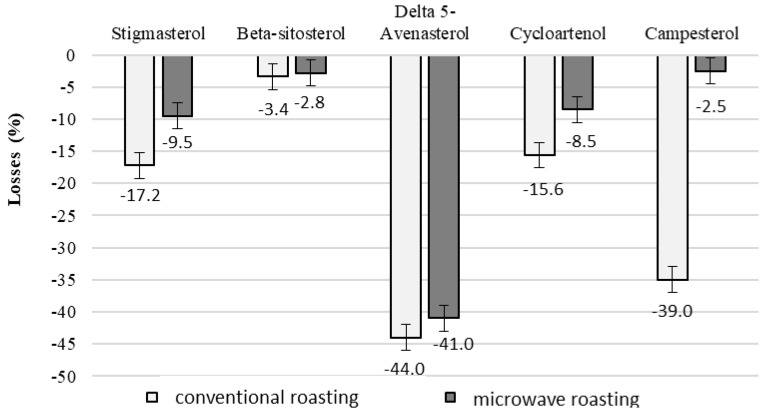
Changes in the content of selected sterols expressed as losses after conventional and microwave roasting of walnuts.

The highest losses after roasting were observed for delta 5-avenasterol (comparable for both roasting methods) (*p* > 0.05), which amounted to over 40%, and the lowest for ß-sitosterol, the losses of which were also comparable for both types of roasting (*p* > 0.05) but were significantly lower and amounted to 3.5% after conventional roasting and 2.8% after microwave roasting (Figure 4).

**Figure 5:**
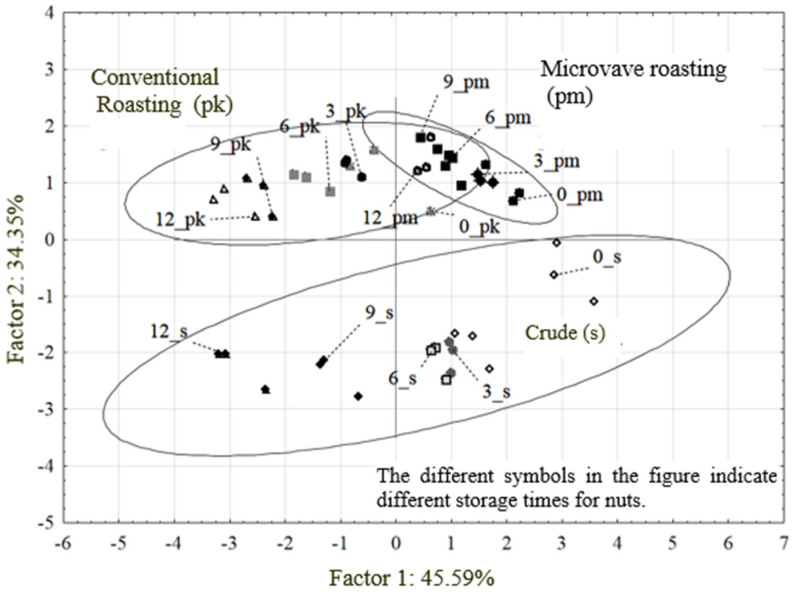
Projection of variables and cases onto the plane of the first two principal components.

Storage time had a different effect on the content of individual sterols. The phytosterol content in raw (crude) and processed (conventional roasting and microwave roasting) nuts stored from 0 to 12 months is presented as a projection of variables and cases (Figure 5).

Microwave roasting showed milder effects on phytosterol losses in nuts. Figure 5 clearly shows that the microwave-roasted (pm), stored samples stand out and are clustered on the same side as the unroasted (raw) samples. It is obvious that the microwave-roasted (pm), stored samples are the most stable.

The first principal component (Factor 1) explains 45.59% of the total variability, and the second principal component (Factor 2) explains 34.5% of the variability. The clustering of samples on the right side of the PCA plot indicates that the phytosterol content is more favorable for nuts that were not roasted and stored for more than 9 months.

The phytosterol content of the microwave-roasted nuts was improved primarily due to the conditions encountered during this processing. Microwave heating is rapid and efficient, which leads to the uniform heating of samples, preventing the formation of excessive local heat sources. Nut samples stored after microwave roasting may be more stable due to the faster roasting process, which minimizes contact with oxygen, moisture and high temperature. This reduces fat oxidation and inhibits unfavorable chemical reactions, including phytosterol oxidation.

## 3. Discussion

Nuts are seen as a very good source of phytosterols, i.e., components commonly used in the treatment and management of diabetes, various malignancies, cardiovascular disorders, atherosclerosis and skin problems [4]. Nuts are consumed in the diet both as raw and roasted products. At home, collected tree nuts are stored in different ways and under different conditions in an annual cycle, i.e., until the nuts are obtained from the next harvest. The same reference is used for obtaining this raw material for industrial processing.

However, there are few data in the available literature differentiating the sterol content in nuts depending on processing or storage. Therefore, the results obtained in this study may have practical significance, especially in human nutrition, for example, for balancing a diet rich in phytosterols, of which nuts may be a source. It is necessary to determine the actual losses of sterols during the processing or long-term storage of products rich in these components.

It is known that any heat treatment of raw materials rich in plant sterols can cause their decomposition or transformation. They are easily oxidized, which results in the formation of unfavorable oxysterols. The higher the concentration of sterols in food, the greater the probability of a high content of their oxygen derivatives in a given product [7 However, no technological guidelines have been developed so far, i.e., it has not been determined what type of heat treatment should be avoided and what type of treatment should be used in the processing of raw materials rich in these components.

In our study, five main types of sterols were found in nuts, namely, campesterol, stigmasterol, ß-sitosterol, delta 5-avenasterol and cycloartenol. Raw nuts contained from 91.87 to 114 mg/100 g of sterols (hazelnuts), while roasted nuts contained from 98.29 to 105.78 mg/100 g. ß-sitosterol was dominant in all types of nuts, but a large amount of cycloartenol was present in walnuts.

Studies conducted by other researchers indicate that the most important types of sterols found in nuts are β-sitosterol, campesterol and stigmasterol [2,27].

Manne et al. [37] indicate that in walnuts, the major form was β-sitosterol, followed by campesterol and delta 5-avenasterol. In hazelnuts, β-sitosterol was the first free sterol in the two samples studied. Other free and esterified sterols, in decreasing order of abundance, were campesterol and delta 5-avenasterol. Other major compounds were saturated sterols, namely, sitostanol and campestanol. In hazelnuts and walnuts, β-sitosterol was found to be the major sterol, and the content of β-sitosterol was higher compared to all other sterols. β-sitosterol also has many applications and is a therapeutically active compound. It is used to lower cholesterol and treat prostate inflammation, but high levels of β-sitosterol in patients suffering from heart attacks lead to serious heart problems.

Gawrysiak-Witulska and Rudzińska [38] and Rudzińska et al. [39] indicate that the content of plant sterols may be significantly influenced by the time and conditions of storage of the raw material. Comparative studies show that rapeseed dried using the low-temperature method shows a decrease in the total amount of sterols by 8–14%, while their annual storage causes a decrease by 15–17%.

The research conducted in this study showed that the sterols contained in raw nuts were systematically lost over 12 months of storage. Depending on the type of nut, they ranged from about 6 to over 20% of the initial amount.

The results obtained in this study indicate that the reduction in sterol content in nuts was dependent on the type of roasting. Roasting resulted in total sterol losses ranging from 7.3 to 13.8%, with microwave roasting causing twice as much loss as conventional roasting.

During storage of the raw nuts (6 and 12 months), the total sterol losses varied for different nuts: 11.5–19.3% for peanuts, 6.8–21.3% for walnuts and 7.2–12.8% for hazelnuts. Microwave roasting proved to be more beneficial in this respect. Nuts roasted in a microwave oven and stored for 12 months lost about 15% less sterols than conventionally roasted nuts. Available scientific studies describe the effect of various technological processes on the content of plant sterols, but such studies were mainly conducted on oils rich in phytosterols.

On the other hand, relatively few studies have been conducted on raw materials rich in phytosterols. This creates the need for extensive studies indicating the content of these valuable components both in raw materials and in the various products produced after processing these raw materials.

According to many authors [40,41,42,43], the degree of degradation of plant sterols in oils is largely influenced by the temperature used during heat treatment. The use of a temperature of 180 °C for 10 min during frying causes a loss of sitosterol content of about 5% in rapeseed oil, to which these phytosterols were added in the form of esters [44].

Kmiecik et al. [45] indicate that 16–19% of phytosterols are lost during frying French fries, but this depends on the type of fried materials. The greatest decrease in phytosterol content is observed during the first 24 h of the long frying process.

In this study, nuts were roasted at 170 °C for up to 20 min, and the total loss of phytosterols was 13.8% of their original amount. Delta 5-avenasterol (walnuts), stigmasterol (hazelnuts) and cycloartenol (peanuts) are the least stable sterols in raw nuts. Stigmasterol (walnuts and peanuts) and ß-sitosterol (hazelnuts) are the most stable. Storage of roasted walnuts after conventional roasting for a longer period (12 months) results in a loss of total sterols of up to 27%, while those roasted by microwave method show only a 5.6% loss of sterols compared to the original content. Stigmasterol is the least stable sterol during the frying process, with losses ranging from 40% to 61% depending on the type of product being fried and the process time [46,47].

Similar results were obtained in the present study. After 6 months of storage of nuts, losses of total phytosterols were 6.8% for walnuts, 7.2% for hazelnuts and 11.5% for peanuts. After 12 months, these losses were 21.3%, 12.8% and 19.3%, respectively. It was also found that during conventional roasting, losses of ß-sitosterol were 3.4%, campesterol 35%, stigmasterol 17.2% and avenasterol over 40%.

## 4. Materials and Methods

### 4.1. Materials

In this study, the following samples were used: hazelnuts (*Corylus avellana*)—country of origin: Azerbaijan, Ata-Baba (Zakatala) variety; common walnuts (*Juglans regia* L.)—county of origin: USA, medium-late Chandler variety and shelled peanuts (*Arachis hypogaea* L.)—country of origin: Nicarague—Runner variety. The samples were obtained by courtesy of the Bakalland S.A. company’s office in the city of Łódź (the company is based in the city of Warsaw, Poland).

### 4.2. Roasting Conditions

Two types of roasting methods were used:

- Conventional—PETRONCINI laboratory nut roaster (Petroncini Impianti S.p.A., Modena, Italy) temp. 170 °C. Due to their diverse structure, the time was adjusted specifically for each type of nut and ranged from 10 to 20 min.
- Laboratory—microwave scale roasting—negative pressure in a microwave–vacuum dryer manufactured by Promis-Tech (PROMIS-TECH Sp. z o.o., Wrocław, Poland), roasting temperature 60 °C, pressure 40 hPa. Due to their diverse structure, the time was adjusted specifically for each type of nut and ranged from 140 to 180 s.

### 4.3. Storage Conditions

Specific samples of raw and roasted nuts were stored in refrigerators (+4 °C) in airtight glass containers without access to oxygen or light.

### 4.4. Methods

#### 4.4.1. Determination of Plant Sterols

Sterol content was determined in fat extracted from nut samples extracted using the Folch method [48]. Sterols were determined using the method described by Derewiaka et al. [49].

The extracted fat sample (approximately 0.2 g) was diluted in 10 mL of hexane and 0.1 mL of a solution of 5α-cholestane and betulin (internal standards for the quantitative determination of sterols (from Sigma-Aldrich, with a declared purity of 98.0% (m/m)) was added. Then, they were saponified for 1 h with 0.5 mL of 1N KOH solution (from Sigma-Aldrich) in methanol (from Sigma-Aldrich) at room temperature. Then, the solvents were removed under a nitrogen stream, and the samples were derivatized by adding 100 µL of anhydrous pyridine (from Sigma-Aldrich, St. Louis, MO, USA). The unsaponifiable fraction was extracted with ethyl ether (from Sigma-Aldrich) and silylated by adding 100 µL of silylating agents (2,2,2-trifluoro-N-trimethylsilyltrimethylethanimidate with 1% trimethylchlorosilane from Sigma-Aldrich, St. Louis, MO, USA). The sterol silane derivatives were dissolved in 1 mL of dichloromethane (from Sigma-Aldrich) and analyzed using a gas chromatograph equipped with FID and ECD detectors Clarus 580 from Perkin Elmer (Shelton, CT United States), coupled to a Perkin Elmer mass spectrometer: Clarus SQ 8T. Perkin Elmer chromatography column marked. Elite 5 ms with stationary phase used: 1,4-bis(dimethylsiloxy)phenylenedimethylpolysiloxane. Column length 100 m, diameter 0.25 mm, film thickness 0.25 µm. Carrier gas: helium with a flow rate of 1.2 mL/min. Analysis was performed at the programmed temperature: initial temperature 80 °C, then increase by 1.5 °C per minute to 250 °C, held at this temperature for 2 min, another increase by 4 °C per minute to 310 °C and held at this temperature for 10 min. After separation of sterols, their quantitative determination was made in relation to the addition of internal standards (betulin and 5α-cholestane) (from Sigma-Aldrich). Identification of sterols was based on comparison of retention time with available standards and mass spectral libraries as well as literature data [50,51]. The detection limit of sterols was from 0 to 0.5 ppm. Measurements were performed three times, and the results were given in milligrams per 100 g of fat. Considering the fat content, the amount of sterols was calculated per 100 g of nuts.

#### 4.4.2. Calculation of Phytosterol Losses After Roasting or Storage

The loss of phytosterols after roasting or storing the nuts was calculated mathematically according to the following formula:

$$losses\;of\;phytosterols=\frac{\left(A-B\right)}{A}\times 100\%$$

in which:

A—Phytosterol content before roasting or storage;

B—Phytosterol content after roasting or storage.

#### 4.4.3. Statistical Methods—Statistical Program Statistica 12.0

The obtained results were statistically analyzed using the Statistica 12.0 computer program. The statistical calculations included the following:

- The arithmetic mean (xmean) and standard deviation (SD);
- The significance of differences between the compared means was tested;
- One-factor and two-factor analysis of variance, designated in the work as ANOVA or MULTI-ANOVA, for an assumed significance level of alpha = 0.05;
- The analysis of principal components (PCA—Principal Component Analysis) was performed in order to determine the dependencies of the obtained results (variables and cases) depending on the storage time, roasting method and type of nuts.

## 5. Conclusions

Five main sterols were identified in the raw and roasted, fresh and stored nuts tested: campesterol, stigmasterol, ß-sitosterol, delta 5-avenasterol and cycloartenol. Significantly more sterols are found in walnuts compared to hazelnuts and peanuts. In all nuts, the dominant phytosterol is ß-sitosterol, and in walnuts, additionally, cycloartenol. The most important phytosterol with the largest share in the total pool of phytosterols in all tested nuts was ß-sitosterol, but in the case of walnuts, it was also cycloartenol, which distinguished walnuts from hazelnuts and peanuts. The total content of phytosterols differed between the nuts depending on the type of heat treatment, but two phytosterols played an important role: delta 5-avenasterol and stigmasterol. The greatest losses of the sterols contained in raw and roasted nuts by both methods occur during the first 6 months of storage. During 12 months of storage, the loss of identified sterols increases, but the rate is slower.

The roasting process causes significant losses of the total phytosterols in nuts, with microwave roasting being more advantageous, after which these losses are almost half those of conventional roasting. Roasting (regardless of the method) causes the greatest losses of delta 5-avenasterol and campesterol and the smallest of ß-sitosterol. Storage of raw nuts reduces the total content of sterols to a different extent for different nuts. After 12 months of storage of conventionally roasted walnuts, the loss of these valuable components is more than twice as high as in the case of nuts previously roasted in a microwave oven. The greatest losses of sterols due to storage are observed for delta 5-avenasterol (in walnuts), stigmasterol (in hazelnuts) and cycloartenol (in peanuts). On the other hand, the lowest losses are observed for stigmasterol (in walnuts and peanuts) and ß-sitosterol (in hazelnuts).

## Figures and Tables

**Table 1:** Phytosterol content in raw nuts (non-stored and after storing).

Type of Sterols Identified	Phytosterol Content [mg/100 g ± SD]		
	Walnuts (*n* = 9)	Peanuts (*n* = 9)	Hazelnuts (*n* = 9)
not stored			
ß-sitosterol	57.2 ± 1.5	50.9 ± 1.2	66.4 ± 0.9
Campesterol	3.8 ± 0.1	9.2 ± 0.7	3.5 ± 0.6
Stigmasterol	2.9 ± 0.3	6.4 ± 0.4	0.8 ± 0.1
Delta 5-avenasterol	5.6 ± 0.2	4.8 ± 0.7	3.6 ± 0.2
Cycloartenol	15.8 ± 0.2	5.3 ± 2.0	4.4 ± 0.1
Total	114.1 ± 1.7	92.7 ± 9.7	91.8 ± 1.6
after 6 months of storing			
ß-sitosterol	54.0 ± 0.3	45.7 ± 1.0	59.8 ± 2.9
Campesterol	3.6 ± 0.1	8.7 ± 0.6	2.6 ± 0.3
Stigmasterol	2.8 ± 0.1	6.3 ± 0.2	0.6 ± 0.1
Delta 5-avenasterol	4.3 ± 0.2	4.5 ± 0.1	2.9 ± 0.1
Cycloartenol	13.6 ± 0.6	4.2 ± 0.4	4.9 ± 0.2
Total	106.3 ± 0.6	82.1 ± 0.6	85.3 ± 2.4
after 12 months of storing			
ß-sitosterol	34.35 ± 1	41.83 ± 1	55.03 ± 2
Campesterol	2.44 ± 1	6.99 ± 1	2.68 ± 1
Stigmasterol	2.14 ± 1	5.91 ± 1	0.11 ± 1
Delta 5-avenasterol	2.71 ± 1	4.19 ± 1	2.68 ± 1
Cycloartenol	11.42 ± 1	3.98 ± 1	4.90 ± 1
Total	89.77 ± 2	74.83 ± 1	80.11 ± 2

**Table 2:** Total phytosterol content of walnuts roasted using two methods and stored for 0 to 12 months in closed glass containers.

Storage Time (Months)	Sterol Content in Roasted Nuts [mg/100 g ± SD]		Losses After Roasting (%)	
	Conventional	Microwave	Conventional	Microwave
Non-Roasted	114.1 ± 2.6			
0—immediately after roasting (*n* = 9)	98.3 ± 3.3 ^a^	105.8 ± 3.1 ^b^	13.8	7.3
3 (*n* = 9)	95.9 ± 1.2 ^a^	103.7 ± 1.3 ^b^	15.4	9.1
6 (*n* = 9)	90.1 ± 2.3 ^a^	100.7 ± 1.2 ^b^	20.6	11.7
9 (*n* = 9)	83.9 ± 2.4 ^a^	100.4 ± 2.2 ^b^	25.9	12.0
12 (*n* = 9)	82.6 ± 1.1 ^a^	99.8 ± 1.1 ^b^	27.2	12.5

Different letter designations in rows indicate significant statistical differences for α = 0.05.

## Data Availability

There are no data outside those reported in this article.
